# Hyalinizing clear cell carcinoma of the nasopharyngeal minor salivary glands: an unusual case report

**DOI:** 10.1097/RC9.0000000000000372

**Published:** 2026-03-10

**Authors:** Sadia Tameez-ud-din, Hafiz Abdul Mughees, Shafiq Ur Rahman, Muneeb Faiz, Irshad Ali, Waseem Sajjad

**Affiliations:** a Department of Medicine, Foundation University Medical College, Islamabad, Pakistan; b Department of Medicine, Saidu Group of Teaching Hospitals/ Saidu Medical College, Swat, Pakistan; c Department of Medicine, King Edward Medical University, Mayo Hospital, Lahore, Pakistan; d Department of ENT, Fauji foundation Hospital, Islamabad, Pakistan

**Keywords:** hyalinizing clear cell carcinoma, immunohistochemistry, nasopharynx, salivary gland tumor, surgical excision

## Abstract

**Introduction and importance:**

This case presents an uncommon instance of hyalinizing clear cell carcinoma (HCCC) of minor salivary glands presented as a mass resembling a nasal polyp in nasal cavities, emphasizing its rarity, diagnostic complexity, and the essential role of immunohistochemistry in accurate identification.

**Case presentation:**

A 15-year-old girl presented in the general outpatient department with progressive nasal congestion for 2.5 years, along with allergic rhinitis, mouth breathing, sleep apnea, and rhinorrhea, further leading to ear blockage and purulent otorrhea. Examination revealed a smooth, lobulated mass on the posterior nasopharyngeal wall extending into the nasal cavity. CT and MRI confirmed a well-defined, homogeneously enhancing 3.4 × 5.0 × 6.4 cm mass. Histopathology showed fibrotic stroma and clear cells. Immunohistochemistry was positive for pan Cytokeratin (AE1/AE3), p63, and CK-7, while Smooth Muscle Antibody, S-100, and GFAP were negative, confirming the diagnosis. The patient underwent endoscopic transpalatal surgical excision with clear margins and recovered well, with no relapse at 1-year follow-up.

**Clinical discussion:**

HCCC is a rare low-grade malignancy with indolent growth but potential for recurrence. Its unusual presentation as a nasal mass often mimics benign conditions, causing delay. Imaging aids evaluation, but immunohistochemistry is vital to distinguish it from other clear cell tumors. Complete surgical excision with negative margins is the treatment of choice, and follow-up is crucial to detect recurrence.

**Conclusion:**

This case highlights the importance of early recognition and accurate diagnosis of HCCC to ensure curative surgical outcomes and prevent metastasis.

## Introduction

Hyalinizing clear cell carcinoma (HCCC) is a rare malignant neoplasm arising from minor salivary glands, particularly the minor salivary glands of the head and neck^[^1^]^. Histologically, HCCC typically presents with tumor cells arranged in sheets, nests, and cords within a hyaline or myxoid stroma^[^2^]^. Normal glandular tissue is absent, and periodic acid-Schiff staining is utilized for diagnosis confirmation^[^3^]^. The cells are polygonal, exhibiting abundant clear-to-eosinophilic cytoplasm, and are often positive for pancytokeratin and CK7^[^4^]^. The presence of EWSR1 rearrangements, particularly EWSR1-ATF1 fusions, is a hallmark of HCCC, aiding in its diagnosis^[^5^]^. HCCCs are generally low-grade malignancies, but they can be locally aggressive and have the potential for distant metastasis^[^6^]^. Here, we report a case of HCCC arising in the nasopharynx in a 15-year-old girl. She presented with a mass resembling a nasal polyp originating from the posterior ala of the nasopharyngeal tube, which after biopsy turned out to be HCCC of minor salivary glands of the nasopharynx. This case report has been written in accordance with the SCARE and TITANS Guidelines^[^7,8^]^.

HIGHLIGHTS

- Hyalinizing clear cell carcinoma of the nasopharyngeal minor salivary glands is an exceptionally rare clinical entity.
- A 15-year-old girl presented with a long-standing nasal obstruction and otologic symptoms mimicking benign conditions.
- Imaging revealed a well-defined nasopharyngeal mass extending into the nasal cavity, initially resembling a nasal polyp.
- Immunohistochemistry confirmed HCCC, highlighting its essential role in differentiating rare salivary gland malignancies.
- Complete surgical excision achieved cure, with no recurrence at one-year follow-up, emphasizing the value of early detection.

## Case presentation

A 15-year-old female presented with a 2.5-year history of progressive nasal congestion, allergic rhinitis, persistent mouth breathing for 1 year, rhinorrhea and sleep apnea. Over the past three weeks, she developed unilateral ear blockage, significantly affecting her quality of life. On history taken by the medical practitioner, the patient had diminished appetite with increased sleep with a profound headache and an ongoing allergic rhinitis. Past medical and socio-economic history was insignificant. Physical examination revealed left-side adenoid hypertrophy associated with Eustachian tube obstruction leading to retraction of tympanic membrane, causing Otitis media with effusion. Further more, a unilateral mass-like nasal polyp was observed arising from the posterior wall of the nasopharyngeal tube and pushing the soft palate anteriorly which then led to certain clinical examinations such as diagnostic endoscopy. All other examinations were unremarkable. There were no signs of respiratory distress or stridor. Certain baseline investigation such as blood CP, serum electrolytes, liver function tests, renal function tests, and serum albumin, was done in which ESR was raised to a value of 29 mm/hour enhancing the susceptibility of inflammation. All other lab findings were insignificant.

**Figure 1.**
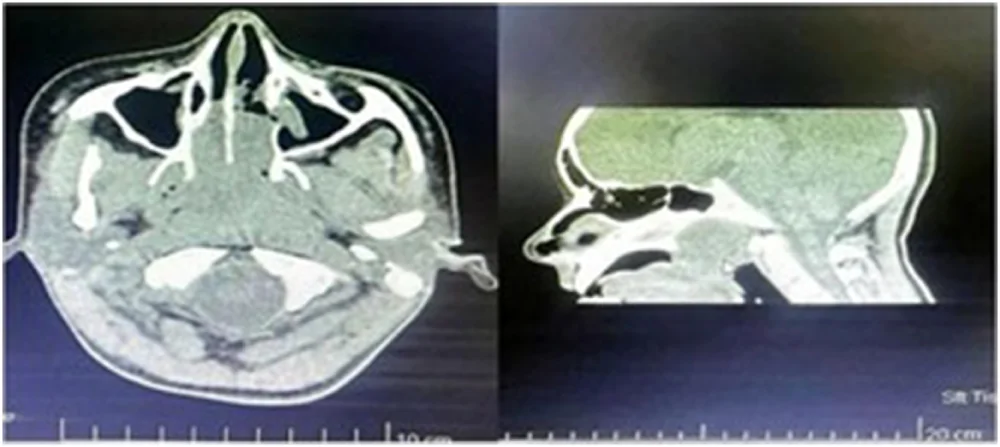
Head CT scan: Left axial CT shows a 3.6 × 3.3 × 3.0 cm nasopharyngeal mass obliterating the nasopharyngeal air columns. Mucosal thickening is noted in the maxillary and ethmoidal sinuses, with air foci in nasal cavities. Right sagittal CT reveals the mass displacing the soft palate inferiorly, causing airway obstruction. Paranasal sinus mucosal thickening is also seen.

**Figure 2.**
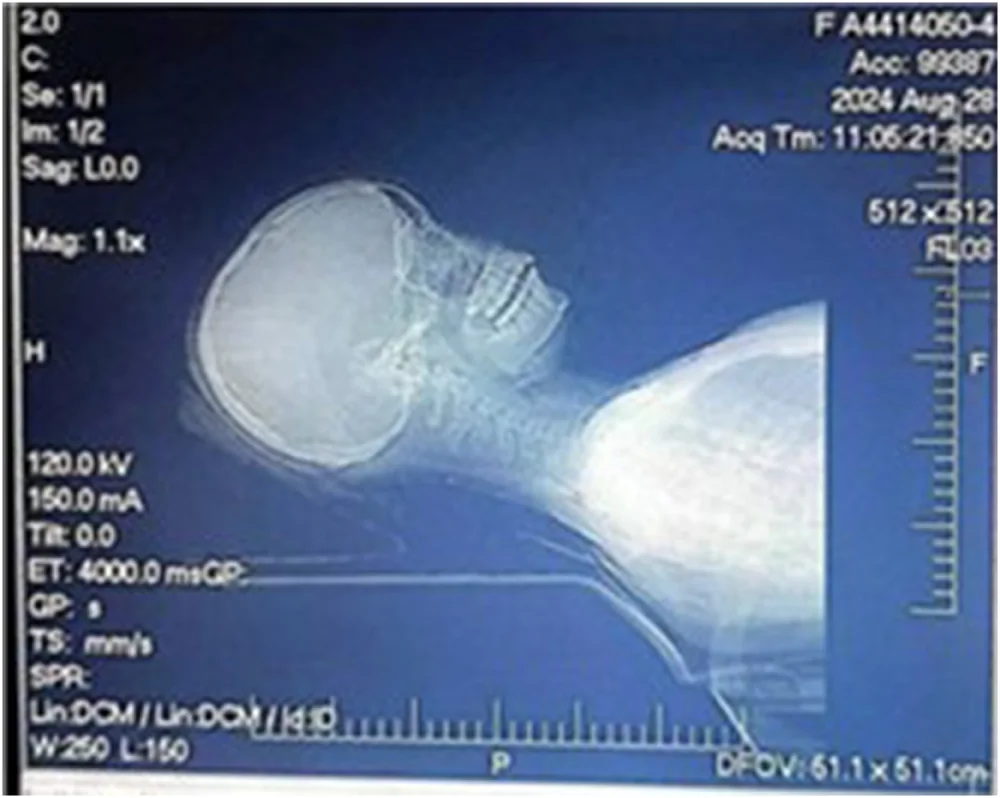
Lateral CT scout image of the head, neck, and upper chest showing the bony outlines of the skull, cervical spine, and upper thorax, with normal alignment of the cervical vertebrae and no obvious gross deformity or large abnormal shadow at this level, outlined with soft tissues.

On clinical examination, transoral and endonasal approached endoscopy revealed a smooth, lobulated mass originating from the posterior nasopharyngeal wall and extending into the nasal cavity. Imaging studies further characterized the lesion, with a CT scan showing a well-defined 3.6 × 3.3 × 3.0 cm mass compressing the soft palate and obstructing the nasopharyngeal air column and posterior nasal choana (Fig. 1). Lateral CT scout image of the head, neck, and upper chest was also performed which showed no obvious gross deformity or large abnormal shadow at this level, outlined (Fig. 2).

**Figure 3.**
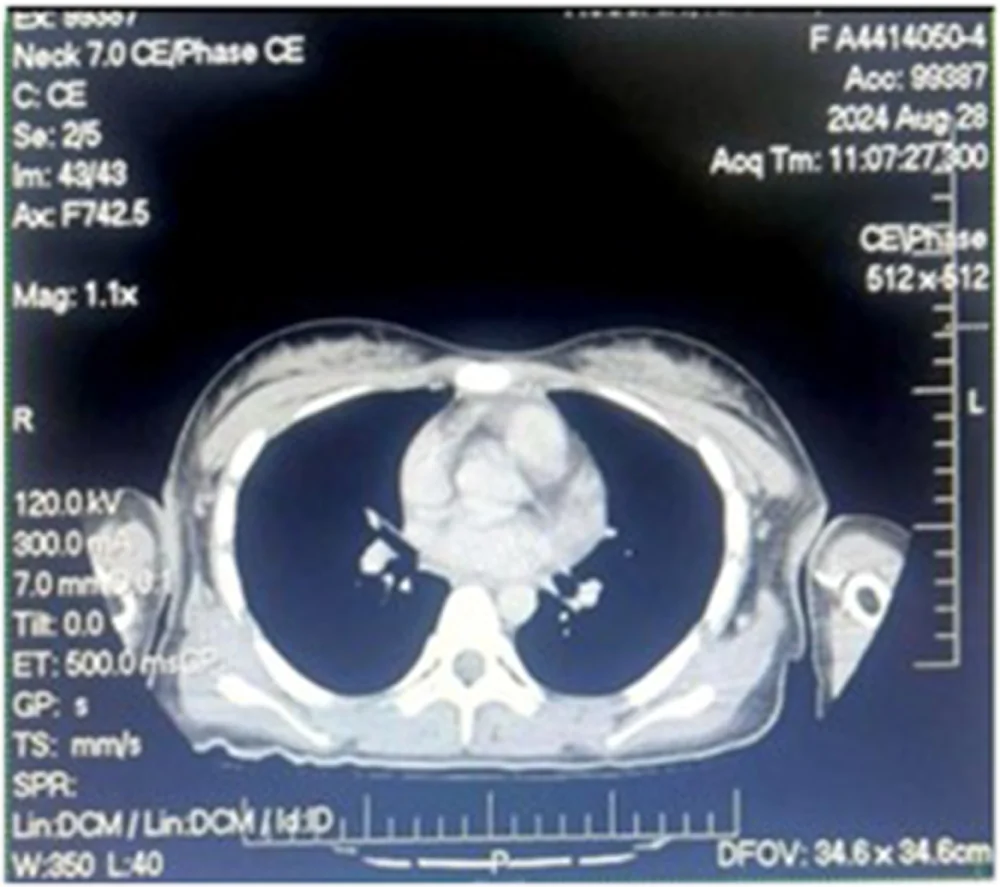
Axial contrast-enhanced CT image of the chest demonstrates both lungs as well-aerated, low-density areas on either side, with the heart and mediastinal structures centrally located and opacified blood vessels due to contrast administration. The cardiac silhouette and major vessels are clearly visualized, the vertebral body is seen posteriorly, and the ribs and chest wall are intact. No obvious large mass, gross consolidation, or significant pleural fluid is apparent.

In addition, to rule out other differentials, Axial contrast-enhanced CT image of the chest was performed which demonstrated well aerated lungs with centrally placed heart and mediastinal structures and no obvious large mass, gross consolidation, or significant pleural fluid is apparent (Fig. 3).

Mucosal thickening in the maxillary and ethmoidal sinuses suggested concurrent acute rhinosinusitis. MRI with Gadolinium contrast, revealed a large, lobulated, homogeneously enhancing 3.4 × 5.0 × 6.4 cm mass. The lesion extended anteriorly into the nasal cavities, causing their obliteration, abutting the floor of the sphenoid sinus superiorly and the lower part of the clivus + first and second vertebral bodies posteriorly. No bony erosion was noted.

Two biopsies were performed under general anaesthesia. The initial biopsy detected a non-specific chronic inflammation without evidence of malignancy. During surgery, the firm mass was excised via a transpalatal approach. Histopathological analysis of the excised specimen revealed fibrotic stroma containing clear cells. Immunohistochemistry confirmed the diagnosis of HCCC with tumor cells showing positive staining for pan-cytokeratin (AE1/AE3), p63, and CK-7, while SMA, S-100, and GFAP were negative, thereby excluding the differential diagnoses of myoepithelioma, myoepithelial carcinoma, and mucoepidermoid carcinoma. The final diagnosis was HCCC of the nasopharynx, a low-grade malignancy with a favorable prognosis following complete excision.

The patient underwent complete tumor excision along with free margins via endoscopic transpalatal approach with no enhancing mass in the nasopharynx. The lesion was meticulously dissected from the posterior soft palate and nasopharyngeal mucosa, achieving total resection without complications. Given the clear surgical margins, no adjuvant chemotherapy or radiotherapy was necessary.

Postoperative recovery was smooth, with minimal morbidity. At the 1-year follow-up, clinical and imaging assessments showed no recurrence or complications. The patient complied with postoperative care and follow-up visits, with no reported adverse effects or unexpected events.

## Discussion

Clear-cell carcinoma (CCC) is an exceptionally rare salivary gland malignancy which primarily affects the palate, the base and sides of the tongue. First reported in 1994 by Milchgrub, it is found predominantly in the intraoral minor salivary glands and elderly females. A literature review reported 254 cases between 1983 and 2020, with the palate as the most common site followed by the tongue^[^9^]^. The symptoms differ depending on the tumor’s site, with the oral region being the frequent location for HCCC. In this case, the patient presented with chief complaints of severe nasal obstruction, nasal polyp and mouth breathing.

The patient was initially managed as a case of nasal polyps, with chronic inflammation, allergic reactions, asthma, infections, immune dysregulation, and other potential differentials comprehensively ruled out. Due to suboptimal therapeutic response and persistent clinical symptoms, further investigations were conducted. Biopsy and immunohistochemistry revealed positivity for pan-CK (AE1/AE3), p63, and CK-7, with negative results for SMA, S-100, and GFAP, confirming HCCC of the minor salivary gland in the nasopharyngeal wall. The tumor was surgically removed via a transpalatal excision, achieving complete resection with tumor-free margins. During a 1-year postoperative follow-up, no signs of recurrence were detected.

The most prevalent indication of nasopharyngeal HCCC is nasal obstruction, which is caused by the tumorous mass obstructing the airway^[^10^]^. Other complaints include otorrhea, foreign body sensation due to tumor expansion in the nasopharynx, epistaxis caused by mucosal involvement, postnasal drip from mucosal irritation, muffled voice or nasal speech, and sometimes tinnitus^[^11^]^. In this case, the patient presented with a 1-year history of gradually worsening nasal congestion, allergic rhinitis, persistent mouth breathing, and continuous rhinorrhea, which significantly affected her daily activities. Over the past 3 weeks, she also developed ear blockage, further contributing to her discomfort. On clinical evaluation, a smooth mass was observed originating from the posterior nasopharyngeal wall, as seen during transoral examination and endonasal endoscopy. The mass extended anteriorly through the posterior nares into the nasal cavities, indicating a locally advanced tumor, with no signs of distant metastasis, consistent with previous studies where distant spread was uncommon in HCCC^[^12^]^. A previous study has reported cases of HCCC originating from the ethmoid and maxillary sinuses, with extension into the nasal cavities. However, this is not always the case. HCCC can also arise from the posterior nasopharyngeal wall, extending into the nasal cavities through the posterior nares in a similar manner^[^13^]^. In our study, a comprehensive evaluation of the oral cavity and tongue revealed no abnormalities. However, previous research has documented cases of HCCC involving the base of the tongue and sublingual region, suggesting that these areas may also serve as potential sites of tumor origin^[^14,15^]^. This cancer can also target the sides of the tongue as the major site of action. Salivary glands beneath the tongue make it a vulnerable site for tumor attack because of its location^[^16^]^.

A CT scan was performed, revealing a well-defined mass measuring 3.6 × 3.3 × 3.0 cm, accompanied by adenoid hypertrophy and obliteration of the nasopharyngeal air column. Subsequently, a contrast-enhanced MRI of the neck, using gadolinium contrast agent, demonstrated a large, well-defined, lobulated, and homogeneously enhancing mass, measuring 3.4 × 5.0 × 6.4 cm, primarily located along the posterior nasopharyngeal wall.

IHC analysis of this case showed positive expression for pan CK (AE1/AE3), p63, and CK-7, while SMA, S-100, and GFAP were negative, thereby excluding myoepithelioma, myoepithelial carcinoma, and mucoepidermoid carcinoma. Additional markers, including CK5, CK8, CK14, CK19, epithelial membrane antigen (EMA), and carcinoembryonic antigen (CEA), which are typically expressed in HCCC, were not investigated. Notably, EWSR1-ATF1 fusion acts as a specific genetic marker for HCCC, aiding in definitive diagnosis; however, molecular testing could not be performed in this case due to limited resources^[^17^]^.

According to the AJCC 8th edition staging system for nasopharyngeal tumors, this lesion was classified as **T1N0M0**. The tumor developed from the posterior nasopharyngeal wall and extended anteriorly into the nasal cavity without bony erosion, parapharyngeal involvement, or skull base invasion, consistent with a T1 tumor stage. There is no cervical lymphadenopathy (N0), or distant metastasis (M0); therefore, the carcinoma was **Stage I**.

The gold standard treatment for stage I HCCC is a complete surgical excision with clear margins. In this case, the patient underwent endoscopic transpalatal surgical resection of the tumor with free margins, and no adjuvant chemotherapy was required as there was no evidence of nodal or distant metastasis on CT and MRI^[^17^]^. The patient followed up for 1 year with no recurrence reported. Notably, chemoradiotherapy alone, without surgical intervention, is insufficient for disease control and is associated with a poor prognosis, potentially worsening the patient’s symptoms rather than providing effective management. Chemoradiotherapy may be considered only in cases of metastatic disease^[^16^]^.

## Limitations

Although the diagnosis of HCCC is supported by a comprehensive immunohistochemical panel including CK5, CK8, CK14, CK19, EMA, and CEA. However, these markers could not be evaluated in this case due to limitations of the laboratory facilities at the institution. Similarly, molecular confirmation via detection of the characteristic EWSR1–ATF1 gene fusion, considered the diagnostic gold standard for HCCC, was not performed because this test is currently available in only a few specialized laboratories. Therefore, the diagnosis was based on the characteristic histopathological features and the limited IHC markers that were available. While these findings were highly indicative of HCCC, the absence of comprehensive IHC and molecular testing is an important limitation. Despite this, the patient has remained recurrence-free, supporting the chosen diagnostic and therapeutic strategy.

## Conclusion

HCCC is an uncommon malignancy of the minor salivary glands, and timely diagnosis plays a critical role in optimizing patient outcomes. Clinicians should consider HCCC in patients presenting with a persistent and abnormal nasopharyngeal mass if initial biopsies reveal some nonspecific inflammatory changes. Accurate diagnosis relies on histopathology combined with immunohistochemistry with typical classical findings including positivity for pan-cytokeratin, CK7, and p63 and negativity for SMA, S-100, GFAP, which is essential for differentiation of HCCC from other clear cell neoplasms. While molecular testing for EWSR1-ATF1 fusions is not always available, it can provide definitive diagnostic confirmation and should be utilized when feasible. Complete surgical excision with clear margins, such as via a transpalatal approach, remains the mainstay of treatment, and regular postoperative follow-ups with imaging is essential in monitoring recurrence, even in low-grade tumors with a favorable prognosis.

## Data Availability

The data that support the findings of this study are available from the corresponding author upon reasonable request.
